# Taxonomy-Based Approaches to Quality Assurance of Ontologies

**DOI:** 10.1155/2017/3495723

**Published:** 2017-10-11

**Authors:** Michael Halper, Yehoshua Perl, Christopher Ochs, Ling Zheng

**Affiliations:** ^1^Informatics Department, New Jersey Institute of Technology, Newark, NJ 07102-1982, USA; ^2^Computer Science Department, New Jersey Institute of Technology, Newark, NJ 07102-1982, USA

## Abstract

Ontologies are important components of health information management systems. As such, the quality of their content is of paramount importance. It has been proven to be practical to develop quality assurance (QA) methodologies based on automated identification of sets of concepts expected to have higher likelihood of errors. Four kinds of such sets (called QA-sets) organized around the themes of complex and uncommonly modeled concepts are introduced. A survey of different methodologies based on these QA-sets and the results of applying them to various ontologies are presented. Overall, following these approaches leads to higher QA yields and better utilization of QA personnel. The formulation of additional QA-set methodologies will further enhance the suite of available ontology QA tools.

## 1. Introduction

Ontologies are central components of biomedical information management systems, where their hierarchies of concept definitions serve to standardize knowledge and facilitate communication. Ontologies have been successfully deployed in data annotation, semantic integration, knowledge discovery, domain vocabularies, and natural language processing, among other areas [1–6]. Given these important roles, it is paramount that the conceptual knowledge within the ontology be accurate, consistent, and as complete as possible with respect to the applications it serves. However, guaranteeing this can be difficult due to the ordinarily extensive size of ontologies, typically on the order of thousands up to hundreds of thousands of concepts, and the concepts' attendant complexity manifested in potentially millions of relationships connecting them. In fact, it is not unusual to find a variety of kinds of errors in a large ontology, including incorrect and omitted IS-A (subsumption) relationships, incorrect lateral (i.e., nonhierarchical) relationships, and erroneous relationship targets.

In this paper, we focus on methodologies for QA of ontologies [7]. Indeed, ontology QA has been a ripe area of research. A review of QA of medical ontologies can be found in [8]. Another review extended the subject material to QA methodologies applied to all forms of controlled biomedical terminologies [7]. QA approaches have targeted a variety of existing ontologies including SNOMED CT [9, 10], the National Cancer Institute thesaurus (NCIt) [11, 12], and the Gene Ontology (GO) [13, 14]. For example, SNOMED CT has been analyzed for its compliance with seven important ontological principles [15]. Adjectival modification was used to assess the consistency of terms in SNOMED CT [16]. A methodology based on Semantic Web technologies was used to judge the consistency of hierarchical and associative relationships in the NCIt [17]. An evolutionary terminology auditing method was applied to gauge the quality of the GO [18].

Specifically, in this paper, we present guidelines successfully employed as the basis for QA techniques that have been derived from *taxonomies*, alternate high-level compact views of ontologies. In previous work (e.g., [19]), the practicality of developing taxonomy-based QA techniques involving the automatic identification of sets of concepts expected to have higher error rates has been demonstrated. There have been two overarching themes to this work: *complex concepts* and *uncommonly modeled concepts*. Intuitively, complex concepts are those that are structurally complex in terms of their numbers of properties and interconnections, which naturally make them more susceptible to erroneous modeling. If a concept looks very different from all other concepts in its neighborhood, or even in the context of the whole ontology, that raises suspicions about its correctness, and we refer to such a concept as “uncommonly modeled” in a subject area. The thread running through these two themes is that concepts of this nature are revealed as outlier sets through the lens of the taxonomies.

A variety of taxonomy-based methodologies have been developed for and utilized in SNOMED CT, the NCIt, and the GO. Additional methodologies have targeted ontologies in the NCBO's BioPortal [20, 21] including the Ontology of Clinical Research (OCRe) [22], the Sleep Domain Ontology (SDO) [23], the Cancer Chemoprevention Ontology (CanCO) [24], and the Ontology for Drug Discovery Investigations (DDI) [25]. And now with a movement toward methodologies encompassing all the ontologies in a family of similar ontologies hosted in the BioPortal [26], it is important to survey the known methodologies that are taxonomy-based so various curators and editors can more readily use them. Arranging them in the context of the guidelines used in their development and thus highlighting existing best practices, it is our expectation that users and researchers will be encouraged to detect more potentially “higher-error” sets that could be the basis for further taxonomy-based QA methodologies.

## 2. Background

The two foundational taxonomies that have been developed are the *area taxonomy* and the *partial-area taxonomy*. These have been utilized with hierarchies in SNOMED CT and the NCIt, among others [27–29]. Variations and extensions to these taxonomies have been deployed in a number of other ontologies, including disjoint partial-area taxonomies [30] and subject-based subtaxonomies [31]. In the following, we present some of the details of each of these two related structures, with examples drawn from the NCIt. A review of taxonomies can be found in the context of a general treatment of ontology abstraction networks in [32]. Let us note that taxonomies have been derived from ontologies in various formats, including OWL [33], OBO format [34], and SNOMED CT RF2 [35].

The area taxonomy is a hierarchically organized graph structure derived automatically from the arrangement of the hierarchical (i.e., IS-A or *subClassOf*) and nonhierarchical (“lateral”) relationships of the concepts in an ontology's hierarchy. It should be noted that the notion of lateral relationship is modeled differently and referred to using various nomenclatures within the context of different ontology representations. For example, it is attribute relationship in SNOMED CT, role in the NCIt, and property restriction in OWL ontologies. In the latter, all stated and inherited property restrictions that appear in equivalence axioms or *subClassOf* axioms are taken to be lateral relationships. In the remainder of this paper, we will use only the generic “IS-A” to refer to a hierarchical relationship; the term “relationship” will strictly be reserved for lateral relationship, unless otherwise noted.

**Figure 1:**
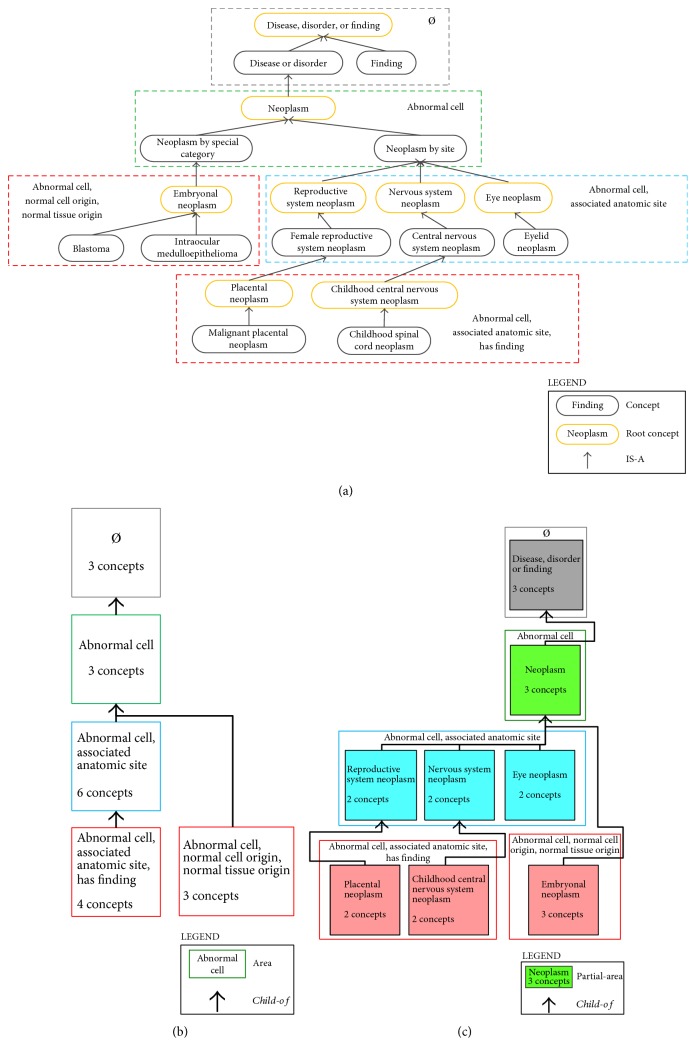
(a) An excerpt of nineteen concepts from NCIt's *Disease, Disorder, or Finding* hierarchy. (b) Area taxonomy excerpt for the concepts in (a). (c) Partial-area taxonomy excerpt for the concepts in (a).

Each node of the area taxonomy graph represents one *area*, defined to be the set of all concepts in the hierarchy exhibiting the exact same set of relationships. The node is labeled with the respective set of relationships and also includes the number of constituent concepts. For example, in the NCIt's *Disease, Disorder, or Finding* hierarchy, there are concepts, such as *Nervous System Neoplasm*, which have the two relationships *Abnormal Cell* and *Associated Anatomic Site*—and only those relationships. (In this example, the “Disease Has” prefix is omitted from all relationship names.) See Figure 1(a), where *Nervous System Neoplasm* and five other concepts with these exact relationships are enclosed in a blue, dashed box. (Not all concepts from this area are shown.) Therefore, there is a node {*Abnormal Cell, Associated Anatomic Site*} indicating six concepts in the area taxonomy—the blue box in Figure 1(b). That figure overall shows the excerpt of five areas from the NCIt's *Disease*, *Disorder, or Finding* area taxonomy corresponding to Figure 1(a).

Nodes of the graph are connected via hierarchical *child-of* relationships, whose derivations are based on the IS-As of the *root* concepts in areas. A root is defined to be a concept at the top of an area's subhierarchy, that is, a concept whose parents all belong to other areas higher up in the ontology's hierarchy. For example, the NCIt concept *Neoplasm by Site* has only the relationship *Abnormal Cell* and thus belongs to the area {*Abnormal Cell*} (Figure 1(a)). *Neoplasm by Site*'s child *Nervous System Neoplasm* is one of the roots of {*Abnormal Cell, Associated Anatomic Site*}. From the IS-A between *Nervous System Neoplasm* and *Neoplasm by Site*, a *child-of* is derived connecting area {*Abnormal Cell, Associated Anatomic Site*} to area {*Abnormal Cell*}. Again, see Figure 1(b) for an illustration.

The area taxonomy is rooted overall in an area with the name “root area.” In figures, such as Figure 1(b), we often display the name as ∅, denoting the fact that its member concepts do not exhibit any relationships whatsoever. (There may be many such concepts, and they may in fact extend deeply into the ontology, as will be discussed further below.) In figures, nodes of the area taxonomy are typically color-coded to indicate their respective levels (equal to their numbers of relationships). For example, the two areas at the bottom of Figure 1(b) have three relationships each and thus have the same color. The root area resides by itself on level 0.

The partial-area taxonomy extends the area taxonomy using local configurations of the IS-As within the ontology. The main difference between the two taxonomies is the inclusion of embedded “subnodes” within area nodes in cases where the area has multiple roots. In such a case, a subnode, called a *partial-area*, is included to represent a concept group comprising one root and all its descendants within its area. Given the fact that the root subsumes all the concepts in its partial-area, its name is used as the label of that group. In the NCIt's *Disease*, *Disorder, or Finding* hierarchy, the area {*Abnormal Cell, Associated Anatomic Site*} has 13 roots (among them are *Reproductive System Neoplasm*, *Nervous System Neoplasm*, and *Eye Neoplasm*).  Figure 1(c) shows an excerpt from the *Disease, Disorder, or Finding* hierarchy's partial-area taxonomy, where the three corresponding partial-areas of {*Abnormal Cell, Associated Anatomic Site*} can be seen. Again, the number of concepts in a partial-area appears beneath its name. *Child-of*s connect partial-areas and are derived from the underlying roots' IS-As in a manner similar to that for areas in the area taxonomy. Certain graphical abridgments are used to reduce the amount of arrows shown [27].

## 3. Methods

The overall goal of automated methodologies for ontology QA is to uncover concepts that are obviously in error or have a high likelihood of being deemed in error on further analysis. Our previous work has sought to identify concepts that have a high likelihood of harboring errors or inconsistencies of different kinds with the taxonomies serving as the guiding frameworks. Effectively, a methodology of this type carries out some kind of analytical extraction of such a set of concepts—which, for example, may already exist in the taxonomy or as an aggregate grouping therein—for a subject-domain expert (e.g., a curator) to review. This saves the person time and effort in focusing their efforts on concepts that are more likely to warrant additional attention. In general, we would say that it enables better utilization of usually limited QA resources and increases the QA yield in terms of discovered ontology problems.

In the context of a given methodology, we refer to the collection of concepts chosen for review as the “Quality Assurance set” (abbreviated “QA-set”). In this section, we introduce four example QA-sets that have been utilized as the basis for QA methodologies. Their utility has been empirically verified in the context of a number of ontologies, some of which are discussed further below in Section 4. Two of the QA-sets are directly based on the area taxonomy. The other two have their origins in the partial-area taxonomy. The four QA-sets are as follows, with the area taxonomy-based ones listed first:

1. The root area
2. The concepts deep in a taxonomy and having many relationships
3. Overlapping concepts
4. Small partial-areas.

In the following subsections, we give the interpretation of each QA-set with respect to the overarching themes of complex concepts and uncommonly modeled concepts. In the last subsection, we introduce some approaches that seek to combine these themes to further enhance QA efforts.

### 3.1. Area Taxonomy-Based QA-Sets

#### 3.1.1. QA-Set 1

As discussed, the root area has concepts having no defined relationships at all. In ontologies, relationships are arguably the most important definitional elements and serve as crucial differentiae [36]. Therefore, it is expectedly uncommon for concepts not to have any relationships to other concepts. In our work, high percentages of concepts without relationships have been located in the root area for some hierarchies. Admittedly, such concepts may have been left underdefined intentionally for concepts that are by nature general or abstract. But one would expect the number to be small. Oftentimes, the omission is unintentional and should be questioned. Hence, the first kind of area taxonomy-based QA-set is the set of concepts belonging to a relatively large root area. The most likely error for such concepts is a missing relationship.

#### 3.1.2. QA-Set 2

As one moves down an ontology hierarchy, there is an expectation of increasing attendant complexity as more and more knowledge is built up—explicitly or implicitly. Most often, the build-up of knowledge in ontologies happens through the introduction of new properties as well as the inheritance of properties from ancestors. The concepts exhibiting increased knowledge aggregation are intuitively more difficult and more subtle to model, and it is natural for errors to more readily occur in such cases. We find a manifestation of this phenomenon in the second kind of QA-set.

The concepts within a taxonomy are organized on numbered levels according to their numbers of relationships. The farther from the root area, the higher the level. Concepts residing deep in the taxonomy, in general, tend to have a large amount of explicit accumulated knowledge, namely, many relationships. With many relationships, there is increased complexity and the chances of erroneous relationships tends to increase. From this, we get the second kind of area taxonomy-based QA-set: levels of the area taxonomy containing concepts exhibiting many relationships.

### 3.2. Partial-Area Taxonomy-Based QA-Sets

#### 3.2.1. QA-Set 3

**Figure 2:**
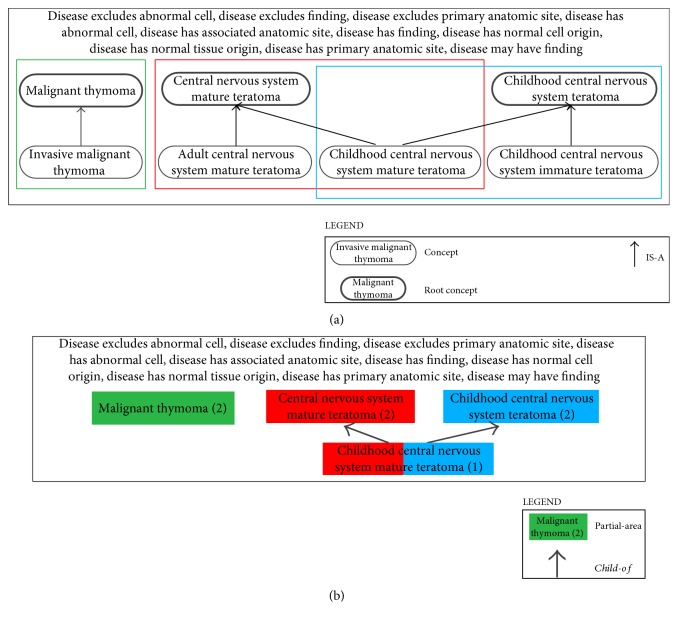
(a) Overlapping concept *Childhood Central Nervous System Mature Teratoma* from the NCIt. (b) Disjoint partial-area taxonomy for (a).

An *overlapping concept* is one that resides in two or more partial-areas within the same area. Such concepts fall under the category of complex concepts due to the fact that they inherit the structure and semantics from two or more partial-area roots. As an example, the NCIt concept *Childhood Central Nervous System Mature Teratoma* resides in the two partial-areas *Central Nervous System Mature Teratoma* and *Childhood Central Nervous System Teratoma* and is thus a descendant of the respective roots. This hierarchical arrangement and grouping into partial-areas is illustrated in Figure 2(a). A QA-set of overlapping concepts is typically quite small, and it is beneficial to review its concepts collectively. In fact, we have developed an additional specialized framework, called the *disjoint partial-area taxonomy* [30], for dealing primarily with this kind of QA-set. The overlapping concepts are extracted from their partial-areas to be in separate “overlapping partial-areas” so that all partial-areas are disjoint. See Figure 2(b) for the corresponding disjoint partial-area taxonomy for Figure 2(a). In Figure 2(b), the overlapping partial-area *Childhood Central Nervous System Mature Teratoma* is drawn as two-colored (red and blue) to denote its concepts' ancestry in the roots of the partial-areas *Central Nervous System Mature Teratoma* (red) and *Childhood Central Nervous System Teratoma* (blue).

#### 3.2.2. QA-Set 4

A small partial-area falls under the category of uncommonly modeled concepts because its concepts exhibit a relatively uncommon combination of structural definitional elements (relationships) and hierarchical locality/grouping. The uncommon combination raises the question of correctness. Small partial-areas have been proven to be useful in uncovering ontology errors because although they may indeed be unusual concepts, it often happens that their uncommon complexity in modeling is due to error. Of course, the threshold value defining the meaning of “small” must be postulated. It has been shown to differ for various ontologies, but it has shown not to stray from a small constant.

### 3.3. Thematic Approaches and Their Combination

Let us note that the common thread running through the two themes of complexity and uncommon modeling is that both kinds of QA-sets consist of outliers in the overall context of an ontology hierarchy. The complex concepts are outliers due to their compound modeling that reflects multiple structural elements and poses challenges for accurate modeling. The uncommonly modeled concepts are outliers by virtue of their unusual structural and/or semantic configurations. It is interesting that these various outliers expressed as QA-sets are not necessarily obvious on inspection of the underlying ontology but surface when the ontology is viewed through the lens of the compact summarizing view afforded by the taxonomies.

The taxonomies are kinds of abstraction networks [32] and as such were not created with the primary purpose of exposing outlier sets that are liable to contain more modeling errors. Our original intent was to employ them in schematically capturing the “big picture” of an ontology network, specifically, its overall content and structure, while ignoring minutiae. Our first forays into abstraction networks had this purpose clearly at the forefront as we formulated object-oriented schemas [37, 38] for the Medical Entities Dictionary (MED) [39] and the Unified Medical Language System (UMLS) [40]. Abstraction work on SNOMED CT led to the creation of the taxonomies as generalizations of the schemas used previously. (See [27] for further details regarding the decision-making processes surrounding the creation of taxonomies. The principal motivation was dealing with less constrained patterns of relationship introductions.)

The realization that abstraction networks in general and taxonomies in particular were excellent platforms for ontology QA came later. Initial—somewhat capricious—discoveries of ontological errors through the lens of schemas and taxonomies made us see this potential. Further analysis revealed that taxonomies tended to cluster the outliers in sets that were statistically more likely to harbor errors. It was concluded that the benefits to QA arose due to the exposure in some instances of concepts of exceptional complexity and, in other instances, concepts of uncommon modeling. This resulted in the QA-sets described above.

Going further, the themes of complexity and uncommon modeling can be combined to enhance the effectiveness of QA regimens and economize the required processing. For example, it may make sense to look at concepts exhibiting complexity from two perspectives (two “layers” of complexity). Or a combination of complex and uncommon characteristics may serve as the basis for additional scrutiny.

One example is the use of the notion of *degree of overlap* as it pertains to overlapping concepts (in the context of QA-set 3). The degree of overlap is defined to be the number of partial-areas to which an overlapping concept belongs. Its value is at least two. As an example, consider the GO concept *ascospore formation*. Since it belongs to the four partial-areas *morphogenesis*, *cell cycle process*, *cell development*, and *reproductive process*, its degree of overlap is four. This characteristic is in fact another layer of complexity for these already complex concepts. So, while overlapping concepts are expected to have more of a likelihood of errors, in general, overlapping concepts of a higher overlap degree are expected to have an even higher likelihood of error vis-a-vis overlapping concepts of a lower degree. The intuition beyond this is an elaboration of additional semantics from more roots and a more involved arrangement of IS-A relationships in the underlying ontology hierarchy—thus, more attendant complexity.

Another example of a combination approach that has yielded significant results is that of concentrating on small partial-areas (QA-set 4) when they appear clustered in an area comprising just a few partial-areas overall—and only small ones at that. In such a circumstance, the small partial-areas, representing concepts with an uncommon semantics and local hierarchical characteristics, are grouped with other such concepts based on an uncommon global characteristic, namely, the area's set of relationships.

An extreme example found originally in the NCIt's *Biological Process* hierarchy is that of the concept *transcription initiation* which resided in a partial-area and an area all by itself [29]. It was the only concept in the whole hierarchy with the relationship combination: *has associated location*, *has result process*, *has result chemical or drug*, and *is part of process*. On analysis, it was deemed to be erroneous, and in a subsequent release of the NCIt, *transcription initiation* no longer had this relationship set; that is, this area disappeared from the area taxonomy. In addition, small partial-areas having concepts with many relationships have been seen as being useful to QA efforts. This latter approach can be viewed as a combination of QA-set 4 with QA-set 2.

A further example is found in the deeper levels of the root area (QA-set 1). With concepts in the root area, there is a build-up of knowledge that is entirely implicit as they do not have any relationship definitions whatsoever. With most of these concepts being primitive—in fact, without having multiple parents, certainly so—one would expect that the internal hierarchical depth of this area to be rather low. If there are concepts that reside a good distance from the hierarchy's root and have no relationships, this raises suspicions: their build-up of knowledge has not been accompanied by the inclusion of any explicit knowledge elements, leaving them likely underdefined. In fact, concepts deep in the root area fall under both categories of complex and uncommonly modeled.

## 4. Results

In this section, we survey some applications of methodologies that are based on the four kinds of QA-sets defined in the previous section. The results of the QA efforts, such as the focus ontology and the scope and kinds of erroneous concepts discovered, are reported. In the Appendix, we include references to supplementary tables that show the error reports from two QA studies. Moreover, the Appendix provides information about accessing the error reports for two additional QA studies at online locations. Let us note that the applications of the described QA methodologies were supported by a number of custom-made software tools, such as OAF [41], which is available as a plug-in for Protégé [42].

### 4.1. Area Taxonomy-Based QA-Sets

#### 4.1.1. QA-Set 1: Root Area

QA-sets comprising the concepts in the root area of an area taxonomy have been the basis of QA analysis done in the context of the NCIt. The focus in that context was on missing-relationship errors, a type of error of omission. In this unpublished study, the root area of the *Biological Process* hierarchy's area taxonomy was seen to be anomalous in terms of its large relative size compared with the rest of the area taxonomy. That hierarchy's root area contained 513 concepts (44.8%) of its total of 1145 concepts. On analysis, it was discovered that 232 of these 513 concepts (45.2%) were missing relationships (referred to as roles in the NCIt). In comparison to a control sample of concepts within the same hierarchy, it was shown that the root area harbored a statistically significantly higher amount of errors using Fisher's exact two-tailed test [43].

Some SNOMED CT hierarchies have been shown to exhibit large root areas, making them suitable for this kind of QA methodology. For example, *body structure* has about 90% of its concepts in its root area, and *event* has 97.5%. Other SNOMED CT hierarchies have root areas that are large in absolute size if not in relative size (i.e., as a percentage of the entire hierarchy). In such cases, they are suitable for a combination QA approach, as discussed in Section 4.3.

#### 4.1.2. QA-Set 2: Concepts Deep in a Taxonomy and Having Many Relationships

This kind of QA-set, with the theme of a division between deeper and shallower levels of the taxonomy, was also used in the context of NCIt's *Biological Process* hierarchy [44]. Those concepts in areas on the deeper levels of the entire area taxonomy, by definition, have more defined relationships than the concepts on shallower levels. In [44], we found that 25% of the analyzed concepts on the levels between level 3 and level 5 (with corresponding numbers of relationships)—with level 5 being the deepest level—were erroneous. This was confirmed as a statistically significantly greater number of errors using Fisher's exact two-tailed test. The errors were verified by S. de Coronado, a manager of the NCIt.

This QA-set was also utilized in the context of the ChEBI ontology [45, 46]. In this unpublished QA study, we asked two chemistry subject-domain experts to formulate a consensus report regarding erroneous concepts throughout the various levels of the taxonomy. At the deepest levels (i.e., levels 5–8), we observed a statistically significantly higher error rate as compared to the shallower levels (as judged again using Fisher's exact two-tailed test). The statistical significance even held as we slightly adjusted the cutoff level between what we considered shallow and deep. The most prevalent types of errors found in this analysis were incorrect lateral relationship targets and incorrect classifications. Modeling problems that were chemistry specific included a number of incorrect charge differences between conjugate acids and conjugate bases and incorrect numbers of cyclic units.

### 4.2. Partial-Area Taxonomy-Based QA-Sets

#### 4.2.1. QA-Set 3: Overlapping Concepts

QA-sets consisting of overlapping concepts were among the focus of QA efforts within the context of SNOMED CT's *Specimen* hierarchy [19], both its 2007 and 2009 versions. In that analysis, it was shown that overlapping concepts are statistically significantly more likely to be erroneous than other concepts. The statistical significance was determined using Fisher's exact two-tailed test. For example, for the 2009 release of the *Specimen* hierarchy, approximately 20% of its concepts were overlapping. Of these, more than half exhibited errors of some kind, including incorrect parents, incorrect relationship types, and incorrect relationship targets. Let us note that the discovered errors were confirmed by K. A. Spackman, who at the time was the Chief Terminologist of the International Health Terminology Standards Development Organisation (IHTSDO)—curator of SNOMED CT. (IHTSDO is now known as SNOMED International.) In general, it was found that overlapping concepts were 1.89 times more likely to be in error than nonoverlapping concepts (55% versus 29%) [19].

Additional analysis within SNOMED CT's *Clinical Finding* hierarchy at the subhierarchy rooted at the concept *Bleeding* showed that overlapping concepts had an error rate that was 4.33 times higher than the nonoverlapping concepts (39% versus 9%) [31]. These results bore statistical significance (using the double bootstrap approach [47]) and were verified by J. T. Case, the acting Chief Terminologist at SNOMED International.

QA-sets of this kind have been utilized within the Gene Ontology (GO) [48], where overlapping concepts were also prone to having a higher error rate than other concepts. Specifically, in GO's *Biological Process* hierarchy, overlapping concepts were 1.39 times more likely to exhibit errors than nonoverlapping concepts (30.2% versus 21.8%). The errors in that study were identified and verified by J. Lomax, who at the time was a coordinator of the GO Editorial Office.

In a study of the overlapping concepts in the NCIt's *neoplasm* subhierarchy, from the *Disease, Disorder, or Finding* hierarchy, a significantly higher number of erroneous concepts (Fisher's exact two-tailed test) were found. Let us note that in this analysis, we only considered errors of a moderate or severe nature, as predefined by the participating subject-domain experts. The error rate for overlapping concepts was found to be 1.6 times that for nonoverlapping concepts (16% versus 10%). Furthermore, the number of errors per erroneous overlapping concept was slightly higher than the number of errors per erroneous nonoverlapping concept (1.33 versus 1.11).

The same phenomenon was observed in the overlapping concepts of Uberon [49], where overlapping concepts had an error rate of 29% versus an error rate of 11% for nonoverlapping concepts [50]. Most of the errors found in this study were confirmed by C. J. Mungall, the curator of Uberon. The results were shown to be statistically significant based on Fisher's exact two-tailed test.

#### 4.2.2. QA-Set 4: Small Partial-Areas

QA-sets of the concepts in small partial-areas were also utilized for SNOMED CT's *Specimen* hierarchy (2004 edition) [27, 28]. Concepts in partial-areas of size seven or fewer were 1.57 times more likely than other concepts to be in error (10.7% versus 6.8%). All the errors reported in [28] were confirmed by K. A. Spackman (the Scientific Director of SNOMED CT at the time and a co-author of the study), and his corrections of the errors were reflected in subsequent SNOMED CT releases.

The *Biological Process* hierarchy of the NCIt underwent a QA review in [29] using this type of QA-set. It was found that 12% of the concepts in partial-areas comprising three or fewer concepts were in error. Overall, such concepts were more than two and a half times more likely to be in error than concepts in the larger partial-areas (12.2% versus 4.6%).

In [51], we analyzed the error rates of concepts in small partial-areas in SNOMED CT's *Procedure* hierarchy. Concepts in small partial-areas were observed to have an error rate of 15.4% versus 8.8% for concepts in large partial-areas. This difference was shown to be statistically significant using Fisher's exact two-tailed test.

Further work on small partial-areas in the context of the NCIt's *neoplasm* subhierarchy was reported in [52]. The observed error rate was twice as large for small partial-areas as compared to large partial-areas. If we considered only the most common kind of error encountered, namely, the missing relationship error, small partial-areas had more than three times the amount of errors.

### 4.3. Combination Approachess

Further analysis within QA-set 3 showed that the degree of overlap (i.e., the number of partial-areas a concept belongs to) is a factor affecting the expectation of erroneousness. We found that the higher the degree of overlap, the higher the error rate. For example, in SNOMED's “*Bleeding*” subhierarchy, concepts that overlapped between two partial-areas had an error rate of 26.7%, three partial-areas, 40.8%, and four or more partial-areas, 64.1% [31]. These results were statistically significant using the double bootstrap technique. In the GO, it was observed that the relatively few concepts with a degree of overlap of four or more had higher error rates (64.7%) compared to other overlapping concepts [48]. J. Lomax of the GO Editorial Office verified the findings.

QA-set 4 was further refined to economize the processing, focusing on small partial-areas clustered tightly in certain areas as well as on small-partial areas whose concepts had many relationships. Applications of these approaches were successfully carried out in SNOMED CT's *Specimen* hierarchy [27]. A collection of errors from this work was reported to K. A. Spackman, SNOMED CT's former Chief Terminologist, who eventually accepted over 90% of them for inclusion in the 2005 release. Further results from this analysis and verification of their statistical significance were presented in [28].

In an approach similar to that in [27], the small partial-areas of the NCIt's *Biological Process* hierarchy appearing together in areas with low numbers of partial-areas in total were closely studied [29]. Hypotheses pertaining to the concentrations of erroneous concepts with respect to these kinds of partial-areas and their aggregations were tested and confirmed.

When the QA-set 1 has significant depth, then those concepts deep in the root area (farthest from its overall root concept) warrant separate attention. For example, analysis of the concepts on the deeper levels within the root area of the NCIt's *Biological Process* hierarchy yielded results showing erroneous concepts occurring at a significantly higher rate than on shallower levels (Fisher's exact two-tailed test). This again was with respect to missing relationship errors. Specifically, three out of every five concepts in the deeper levels of the root area of *Biological Process* were found to be erroneous. We observed a similar trend in GO, where 22.5% of concepts at deeper levels had errors [48].

This combination approach is particularly pertinent when the root areas of hierarchies are large in absolute size, not just relative size. In such cases, a full review of the root area is impractical. A practical approach is to focus on the root area's deeper levels with a higher expectation of discovering and resolving problems. Examples of where this combination approach could be applied are SNOMED CT's *Clinical Finding* and *Procedure* hierarchies. *Clinical Finding* has a root area with 7000 concepts, which is only 6.7% of the entire hierarchy (nearly 104,000 concepts) in release 20160131. *Procedure*'s root area consists of 2591 concepts, 4.7% of its approximately 55,000 concepts. In both cases, the root areas are too large, and a narrowing down of the scope of the QA review is required.

## 5. Discussion

Taxonomy-based QA methodologies using automated QA-set identification have successfully been brought to bear on a number of ontologies. The amount of time and effort expended by domain-expert QA personnel has been reduced with the use of the automated assistance in locating problematic ontology regions. Corrections suggested by our work have effected changes in some very important ontologies used worldwide, including SNOMED CT, the NCIt, ChEBI, and GO.

Moreover, by demonstrating the power and possibilities offered by existing QA-sets, we expect the identification of further QA-sets to expand the suite of tools that can be brought to bear for ontology QA. In particular, the prospects of the design of hybrid QA-sets, as illustrated in Sections 3.3 and 4.3, are exciting since such hybrid techniques seem to offer higher error yields when they have been successfully applied thus far.

Ontologies are human-made representations of knowledge. As such, modeling errors in ontologies are caused by human factors. Thus, when designing QA methodologies to identify sets of concepts that have relatively higher error rates, we are speculating about where the ontology designers and editors are more likely to commit such modeling errors. It is noteworthy that such concepts are outliers, detectable as such not in the original ontology structure but in the alternate compact view afforded by the taxonomy. In particular, those outliers, as we elaborated above, are the QA-sets that follow the two themes introduced in this paper, complex concepts and uncommonly modeled concepts. Those QA-sets were shown to harbor modeling errors in a variety of ontologies. Various interpretations of “complex” and “uncommonly modeled” were illustrated. It is interesting that, when considering hybrid QA-sets with compound characterizations, the combination of reasons for errors is manifested by a higher error rate compared to QA-sets based on only one characterization. In other works on QA of the MED [39], we have identified a QA-set consisting of intersection classes with small extents in the abstraction network obtained as an object-oriented database schema of the MED [37], another example of a hybrid QA-set combining again the theme of complex concept and uncommon modeling in a different context. We observe that QA-sets constituting hybrid combinations of these two themes seem to recur across a diverse range of terminological contexts.

We note that the studies described in this paper used partial-area taxonomies created from the inferred relationships of each ontology (i.e., after a reasoner had been applied). This was intentional, as most end users interact with the inferred version of an ontology, and their applications will accordingly be affected by the errors in the inferred relationships. This also means that the QA characteristics described in this paper were based on the inferred version of the ontology only. Furthermore, the errors reported in our previous QA studies, which were used to establish the error rates for the various characteristics, were errors in the inferred relationships.

Correcting errors in an ontology's inferred relationships typically requires modifying its stated relationships. The stated corrections needed to fix a given error may not be apparent based on the type of error found in the inferred relationships. For example, a missing parent in the inferred version of an ontology may be caused by a missing relationship in the stated relationships (and thus, the reasoner was not able to infer the correct parent) rather than there being a missing stated parent. Indeed, in [53], it was shown that taxonomies help to expose errors that cannot be detected automatically by classifiers. In [31], we identified stated relationship errors that caused errors in the inferred relationships of overlapping concepts. We plan to further investigate the association between the characteristics described in this study and the errors in the stated modeling of ontologies in a future study.

After a QA analysis is completed and the erroneous concepts have been corrected, several possibilities exist regarding the characteristics that drew attention to those concepts in the first place. Ideally, each corrected concept will no longer exhibit any such characteristics. However, the possibility exists for a concept to still exhibit the same characteristic after it has been corrected (e.g., an overlapping concept may still be an overlapping concept if the only error identified and corrected was an incorrect relationship) or exhibit an entirely different characteristic (e.g., removing an erroneous relationship from a concept that has only one relationship will cause the concept to move to the root area).

This also raises the question of the value of our characteristic-based predictions once errors are reported and addressed. Interestingly, after 20 years of research on the QA of ontologies, we have not encountered a situation where all of the errors in an ontology were corrected and the ontology is entirely error free. On the contrary, when we repeated the QA analysis of overlapping concepts in SNOMED CT's *Specimen* hierarchy in 2004 and 2009, we found that the 2009 version had as many erroneous concepts as the 2004 version did (though the errors were not the same). One reason was that when SNOMED CT editors corrected the previously reported errors, they unwittingly introduced new errors. Another reason was that when new concepts were introduced to the ontology, they were introduced with modeling errors. Thus, even though the errors in the overlapping concepts in the 2004 release were corrected, and some of those concepts were still overlapping concepts, new erroneous concepts exhibited this characteristic in the 2009 release.

### 5.1. Limitations

A limitation of the approach is that the target ontology be amenable to the derivation of a taxonomy. The original methodology was designed for certain DL-based ontologies (terminologies) like SNOMED CT and the NCIt. For other ontologies, ad hoc adaptations had to be employed. However, focusing on ontologies in families of similar ontologies [26] in the BioPortal has made the automatic generation of taxonomies [41] possible across a wide spectrum of ontologies.

The specific QA-sets presented herein are expected to help a wide range of curators and editors more effectively deal with the paramount concern of QA in their ontologies. However, we point out that not every methodology is applicable to every ontology, even if that ontology has the structural characteristics required for the creation of an area taxonomy or a partial-area taxonomy underlying the methodology. For example, for QA-set 1, not every ontology hierarchy has a large root area. In the NCIt, the *Gene Product* hierarchy is such an example.

For QA-set 2, an ontology may have only a small number of concepts exhibiting many relationships. Thus, QA analysis of such concepts with an expectation of higher error rates may not be a practical approach for that ontology.

Consider, for example, the case of the *neoplasm* subhierarchy of the NCIt's *Disease, Disorder, or Finding* hierarchy. It has 8166 concepts and due to its importance to the mission of NCI, the quality of their modeling is of high priority to the NCIt curatorial team (S. de Coronado, NCIt manager, personal communication). Due to the large number (27) of relationships available for neoplasm concepts, there are 4824 partial-areas, and 6581 concepts (81% of the total) belong to small partial-areas for which our QA study has shown higher error rates.

On the other hand, the number of overlapping concepts in this hierarchy is relatively low, only 225 concepts (2.7%). The reason for this low number is again the high number of potential relationships, dividing the concepts into many relatively small areas and partial-areas. In the analysis of all these 225 overlapping concepts, they were shown to have on the average statistically significantly more errors than concepts in a control sample. But there are no more overlapping concepts to review, based on this knowledge. In contrast, in the GO, there are many overlapping concepts.

Hence, one should view the various methodologies described in this paper as a collection of tools in a toolkit. One should choose to apply for each ontology the proper approaches that will enable the correction of many concepts per a given effort of review. In some cases, there are several applicable methodologies for the same ontology.

There are of course exceptions. For example, in the *Gene* hierarchy of the NCIt, all the gene concepts reside in singletons, that is, partial-areas of size one. Hence, these concepts are not outliers in this context, and the technique of reviewing concepts in small partial-areas does not offer any advantage.

The general QA-set framework has been shown to be effective in carrying out ontology QA. Our purpose in this paper is to have ontology QA professionals become familiar with the various QA-sets so that they can tailor the proper QA toolkits according to the properties of the ontology hierarchies they are dealing with.

## 6. Conclusions

It has proven to be practical to develop QA techniques based on the automated extraction of sets of concepts (“QA-sets”) that are expected to have higher error rates. Taxonomies, kinds of ontology abstraction networks, have been shown to be excellent frameworks for the identification of such QA-sets based on various structural features. In this paper, we discussed different methodologies for identifying four kinds of QA-sets. Some applications to existing ontologies were presented. The methodologies were organized around two basic themes, the notions of complex concept and uncommonly modeled concept. Overall, following such approaches leads to an enhanced suite of ontology QA tools and better utilization of QA personnel. It is expected that the work presented herein will inspire the formulation of additional QA-sets to further aid efforts in the all-important task of ontology QA and, in particular, to promote hybrid techniques combining multiple aspects that can raise the error-discovery yield.

## Supplementary Material

Supplementary Table 1: Error data set from a QA analysis of NCIt *Biological Process* concepts. Supplementary Table 2: Error data set from a QA analysis of NCIt *neoplasm* concepts.

## Appendix

We have provided two supplementary tables showing the error data sets resulting from the studies in [44] and [52], respectively. The first table, labeled Supplementary Table 1 available online at https://doi.org/10.1155/2017/3495723 and consisting of three columns, lists NCIt *Biological Process* concepts along with the errors discovered with respect to a QA-set 2 analysis. Also included in the table is the number of role (relationship) types exhibited by each concept. The second, two-columned table (Supplementary Table 2) shows NCIt *neoplasm* concepts and their errors found in a QA-set 4 study. Additionally, the results of the combination QA approach described in [31] are available online (https://uscrs.nlm.nih.gov) on the NLM's SNOMED CT U.S. Content Request System (USCRS) under Batch ID 121149. To access the USCRS, login credentials for the NLM's UMLS Terminology Services are required. Also available online (https://github.com/obophenotype/uberon/issues/1243) is the error report for the QA-set 3 approach applied to Uberon [50], along with responses from Uberon's curators.
