# Supplementary material for: Taxonomy-Based Approaches to Quality Assurance of Ontologies
Source: J Healthc Eng. 2017 Oct 11;2017:3495723. doi: 10.1155/2017/3495723 (PMC5660792; doi:10.1155/2017/3495723)
Supplement: Supplementary file 1 — Supplementary Table 1: Error data set from a QA analysis of NCIt Biological Process concepts. Supplementary Table 2: Error data set from a QA analysis of NCIt neoplasm concepts. [file 3495723.f1.pdf]

Supplementary Table 1

NCIt Biological Process Concept	# Role Types	Error
Excision Repair	1	Missing the role Biological_Process_Has_Associated_Location with the target concept Chromosome
Expiration	1	Missing the role Biological_Process_Has_Associated_Location with the target concept Lung
Homologous Recombination	1	Missing the role Biological_Process_Has_Associated_Location with the target concept Chromosome
Mismatch Repair	1	Missing the role Biological_Process_Has_Associated_Location with the target concept Chromosome
Oncogene Activation Process	1	Missing the role Biological_Process_Has_Associated_Location with the target concept Chromosome
Recombination Repair	1	Missing the role Biological_Process_Has_Associated_Location with the target concept Chromosome
Tumor Immunity	1	The target of the role Biological_Process_Has_Result_Biological_Process Cancer Progression is incorrect
Epithelial Cell Proliferation	2	Missing the role Biological_Process_Has_Result_Anatomy with the target concept Epithelial Cell
Nervous System Development	2	Missing the role Biological_Process_Has_Associated_Location with the target concept Nervous System
Telomere Recombination	2	Missing the role Biological_Process_Has_Associated_Location with the target concept Chromosome
Erythrocyte Differentiation	3	Missing the role Biological_Process_Has_Associated_Location with the target concept Bone Marrow
Leukocyte Differentiation	3	Missing the role Biological_Process_Has_Associated_Location with the target concept Bone Marrow
Leukopoiesis	3	Missing the role Biological_Process_Has_Associated_Location with the target concept Bone Marrow
Megakaryocyte Differentiation	3	Missing the role Biological_Process_Has_Associated_Location with the target concept Bone Marrow
Megakaryopoiesis	3	Missing the role Biological_Process_Has_Associated_Location with the target concept Bone Marrow<br>Missing the role Biological_Process_Has_Result_Anatomy with the target Megakaryocyte
Myeloid Cell Differentiation	3	Missing the role Biological_Process_Has_Associated_Location with the target concept Bone Marrow<br>Missing the role Biological_Process_Has_Result_Anatomy with the target Megakaryocyte
Myelopoiesis	3	Missing the role Biological_Process_Has_Result_Anatomy with the target concept myelocyte

NCIt Biological Process Concept	# Role Types	Error
T-Cell Activation	3	Missing the role Biological_Process_Has_Result_Biological_Process with the target concept T Cell Proliferation
Thrombocyte Differentiation	3	Missing the role Biological_Process_Has_Associated_Location with the target concept Bone Marrow
Anaphase	4	The role Biological_Process_Is_Part_Of_Process with the target concept Cell Cycle Process is incorrect
Antigen Presentation	4	The role Biological_Process_Is_Part_Of_Process with the target concept Immune Response Process is incorrect
Cell Cycle Regulation Process	4	The role Biological_Process_Is_Part_Of_Process with the target concept Cell Cycle Process is incorrect
Chromosome Segregation	4	The role Biological_Process_Is_Part_Of_Process with the target concept Cell Cycle Process is incorrect
Cytokinesis	4	The role Biological_Process_Is_Part_Of_Process with the target concept Cell Cycle Process is incorrect
Metaphase Process	4	The role Biological_Process_Is_Part_Of_Process with the target concept Cell Cycle Process is incorrect
Mitosis	4	The role Biological_Process_Is_Part_Of_Process with the target concept Cell Cycle Process is incorrect
Mitotic Chromosomal Process	4	The role Biological_Process_Is_Part_Of_Process with the target concept Cell Cycle Process is incorrect
Mitotic Sister Chromatid Cohesion	4	The role Biological_Process_Is_Part_Of_Process with the target concept Cell Cycle Process is incorrect
Negative Regulation of G0 to G1 Transition	4	Missing the role Biological_Process_Has_Result_Biological_Process with the target concept G1 Phase Process<br>The role Biological_Process_Is_Part_Of_Process with the target concept Cell Cycle Process is incorrect
Negative Regulation of G1 Phase	4	The role Biological_Process_Is_Part_Of_Process with the target concept G1 Phase Process is incorrect
Negative Regulation of G2 Phase	4	The role Biological_Process_Is_Part_Of_Process with the target concept Cell Cycle Process is incorrect
Negative Regulation of Mitosis	4	The role Biological_Process_Is_Part_Of_Process with the target concept Cell Cycle Process is incorrect
Negative Regulation of S Phase	4	The role Biological_Process_Is_Part_Of_Process with the target concept Cell Cycle Process is incorrect
Nuclear Division	4	Missing the role Biological_Process_Has_Associated_Location with the target concept Nucleus

NCIt Biological Process Concept	# Role Types	Error
Positive Regulation of Mitosis	4	The role Biological_Process_Is_Part_Of_Process with the target concept Cell Cycle Process is incorrect
Prophase Process	4	The role Biological_Process_Is_Part_Of_Process with the target concept Cell Cycle Process is incorrect
S Phase Process	4	The role Biological_Process_Is_Part_Of_Process with the target concept Cell Cycle Process is incorrect
Sister Chromatid Exchange Process	4	The role Biological_Process_Is_Part_Of_Process with the target concept Cell Cycle Process is incorrect
Negative Regulation of G1 to S Transition	5	The role Biological_Process_Is_Part_Of_Process with the target concept G1 to S Transition Process is incorrect
Negative Regulation of G2 to M Transition	5	The role Biological_Process_Is_Part_Of_Process with the target concept G2 to M Transition Process is incorrect

Supplementary Table 2

NCIt Neoplasm Concept	Error
Benign Adrenal Medulla Neoplasm	Missing the role Disease_Has_Finding with the target Indolent Clinical Course
Benign Epithelial Neoplasm	Missing the role Disease_Has_Finding with the target Benign Cellular Infiltrate
Gangliocytoma	Missing the role Disease_Excludes_Finding with the target Poorly Differentiated Lesion
Intramuscular Myxoma	Missing the role Disease_Has_Finding with the target Hypocellular Tissue
Lymphoplasmacyte-Rich Meningioma	Missing the role Disease_Has_Primary_Anatomic_Site with the target Meninges
Anal Canal Neuroendocrine Tumor	Missing the role Disease_Has_Normal_Tissue_Origin with the target Neuroendocrine Tissue
Benign Urethral Neoplasm	Missing the role Disease_Has_Normal_Tissue_Origin with the target Connective and Soft Tissue
Cutaneous Glomangioma	Missing the role Disease_Excludes_Abnormal_Cell with the target Malignant Cell
Cervical Intraepithelial Neoplasia	Missing an IS-A link to the concept Premalignant Neoplasm

NCIt Neoplasm Concept	Error
Fibromatosis	Missing the role Disease_Has_Primary_Anatomic_Site with the target Connective and Soft Tissue
Hemolymphangioma	Missing the role Disease_Has_Associated_Anatomic_Site with the target Lymphatic Vessel
High Grade Esophageal Squamous Intraepithelial Neoplasia	Missing the role Disease_Has_Finding with the target High Grade Lesion
Malignant Exocrine Pancreas Neoplasm	Missing the role Disease_Excludes_Finding with the target concept Favorable Clinical Outcome
Ampullary Noninvasive Pancreatobiliary Papillary Neoplasm with Low Grade Dysplasia	Missing the role Disease_Has_Finding with the target Low Grade Lesion
Benign Iris Neoplasm	Missing the role Disease_Has_Finding with the target Benign Cellular Infiltrate
Borderline Ovarian Clear Cell Adenofibroma	Missing the role Disease_Mapped_To_Gene with the target ARID1A Gene
Breast Carcinoma with Osseous Metaplasia	Missing the role Disease_Has_Finding with the target Mixed Cellular Population
Grade 2 Immature Ovarian Teratoma	Missing the role Disease_Has_Normal_Cell_Origin with the target Neuroepithelial Cell
High Grade Vaginal Intraepithelial Neoplasia	Missing the role Disease_Has_Primary_Anatomic_Site with the target Vagina
Invasive Hydatidiform Mole	Missing the role Disease_Has_Primary_Anatomic_Site with the target Uterus
Breast Tubular Adenoma	Missing an IS-A link to the concept Tubular Adenoma
Gonadoblastoma	Missing an IS-A link to the concept Neoplasm, Uncertain Whether Benign or Malignant
Jugulotympanic Paraganglioma	Missing an IS-A link to the concept Neoplasm, Uncertain Whether Benign or Malignant
Adenocarcinoma in Multiple Adenomatous Polyps	Missing the role Disease_Has_Associated_Disease with the target Polyposis
Anterior Tongue Adenoid Cystic Carcinoma	Missing the role Disease_Has_Finding with the target Slow Growing Mass
BCLC Stage B Hepatocellular Carcinoma	Missing the role Disease_Has_Finding with the target Solid Growth Pattern

NCIt Neoplasm Concept	Error
BCLC Stage C Hepatocellular Carcinoma	Missing the role Disease_Has_Finding with the target Solid Growth Pattern
Granulosa Cell Tumor	Missing the role Disease_Has_Normal_Cell_Origin with the target Granulosa Cell
Hemangioendothelioma	Missing an IS-A link to the concept Neoplasm, Uncertain Whether Benign or Malignant
Acantholytic Squamous Cell Skin Carcinoma	Missing the role Disease_Excludes_Abnormal_Cell with the target Malignant Basaloid Cell
Alveolar Rhabdomyosarcoma	Missing the role Disease_Excludes_Primary_Anatomic_Site with the target Bone
Amelanotic Melanoma	Missing the role Disease_Excludes_Finding with the target Favorable Clinical Outcome
Benign Cecum Neoplasm	Missing the role Disease_Has_Finding with the target Indolent Clinical Course
Clear Cell Squamous Cell Skin Carcinoma	Missing an IS-A link to the concept Primary Malignant Neoplasm
Colon Carcinoma by AJCC v7 Stage	Missing the role Disease_Excludes_Finding with the target Favorable Clinical Outcome
High Grade Prostatic Intraepithelial Neoplasia	Missing the role Disease_Has_Finding with the target High Grade Lesion
High Grade Prostatic Intraepithelial Neoplasia, Inverted Variant	Missing the role Disease_Has_Finding with the target High Grade Lesion
Benign Buccal Mucosa Neoplasm	Missing the role Disease_Has_Finding with the target Indolent Clinical Course
Benign Floor of the Mouth Neoplasm	Missing the role Disease_Has_Normal_Tissue_Origin with the target Connective and Soft Tissue
Benign Hard Palate Neoplasm	Missing the role Disease_Has_Finding with the target Benign Cellular Infiltrate
Benign Posterior Tongue Neoplasm	Missing an IS-A link to the concept Benign Soft Tissue Neoplasm
Colorectal Carcinoma by AJCC v7 Stage	Missing the role Disease_Excludes_Finding with the target Favorable Clinical Outcome
Colorectal Signet Ring Cell Carcinoma	Missing the role Disease_Excludes_Finding with the target Favorable Clinical Outcome

NCIt Neoplasm Concept	Error
Elastofibroma	Missing the role Disease_Has_Primary_Anatomic_Site with the target Elastic Fiber
Fibrous Hamartoma of Infancy	Missing the role Disease_Has_Finding with the target Dermal Involvement
Invasive Micropapillary Breast Carcinoma	Missing the role Disease_Has_Finding with the target Aggressive Clinical Course
Appendix Tubular Carcinoid	Missing the role Disease_Has_Normal_Tissue_Origin with the target Neuroendocrine Tissue
Benign Cerebellar Neoplasm	Missing the role Disease_Has_Finding with the target Benign Cellular Infiltrate
Benign Cerebral Neoplasm	Missing the role Disease_Has_Finding with the target Benign Cellular Infiltrate
Benign Skeletal Muscle Neoplasm	Missing the role Disease_Has_Associated_Anatomic_Site with the target Skeletal Muscle Tissue
Benign Supratentorial Neoplasm	Missing the role Disease_Excludes_Primary_Anatomic_Site with the target Infratentorial Brain
Bone Marrow Neoplasm	Missing the role Disease_Has_Primary_Anatomic_Site with the target Bone Marrow
Clear Cell Adenoma	Missing the role Disease_Excludes_Abnormal_Cell with the target Malignant Cell
Intraductal Papilloma	Missing the role Disease_Excludes_Finding with the target concept Invasive Lesion
Malignant Childhood Hemangiopericytoma	Missing the role Disease_Has_associated_Anatomic_Site with the target concept Vascular System
Malignant Esophageal Neoplasm by Anatomic Region	The semantic type Neoplastic Process is wrong
Acute Erythroid Leukemia	Missing the role Disease_Excludes_Primary_Anatomic_Site with the target Lymphatic System
Acute Myelomonocytic Leukemia	Missing the role Disease_Excludes_Primary_Anatomic_Site with the target Lymphatic System
Benign Fallopian Tube Neoplasm	Missing the role Disease_Has_Finding with the target Benign Cellular Infiltrate

**NCIt Neoplasm Concept**	**Error**
Colon Adenocarcinoma	Missing the role Disease_Has_Finding with the target Favorable Clinical Outcome
Colon Adenoma	Missing the role Disease_Has_Finding with the target Favorable Clinical Outcome
Colorectal Adenoma with Moderate Dysplasia	Missing the role Disease_Excludes_Abnormal_Cell with the target Malignant Cell
Colorectal Adenoma with Severe Dysplasia	Missing the role Disease_Excludes_Abnormal_Cell with the target Malignant Cell
Human Papillomavirus-Related Malignant Neoplasm in AIDS Patient	Missing the role Disease_Has_Associated_Disease with the target Acquired Immunodeficiency Syndrome
Intraductal Breast Neoplasm	Missing the role Disease_Has_Finding with the target Intraductal Lesion
Iris Spindle Cell Melanoma	Missing the role Disease_Has_Finding with the target Melanin Pigment Present
Lobular Neoplasia	Missing an IS-A link to the concept Neoplasm, Uncertain Whether Benign or Malignant
Intraventricular Brain Neoplasm	Missing a child concept Glioblastoma
Gallbladder Mucinous Cystic Neoplasm	Missing the role Disease_Has_Primary_Anatomic_Site with the target Digestive System
Gallbladder Sarcoma	Missing the role Disease_Has_Primary_Anatomic_Site with the target Digestive System
Adenocarcinoma with Spindle Cell Metaplasia	Missing the role Disease_Has_Abnormal_Cell with the target Malignant Spindle Cell
Combined Carcinoid and Adenocarcinoma	Missing an IS-A relationship to the concept Cacinoid tumor
Malignant Endometrial Neoplasm	Missing the role Disease_Excludes_Abnormal_Cell with the target Neoplastic Smooth Muscle Cell
Reproductive Endocrine Neoplasm	Missing an IS-A relationship to the concept Endocrine Neoplasm
Tonsillar Carcinoma	Missing the role Disease_Has_Associated_Anatomic_Site with the target Throat
Skull Neoplasm	Missing an IS-A relationship to the concept Bone Neoplasm
